# Signatures of natural selection on genetic variants affecting complex human traits^☆^

**DOI:** 10.1016/j.atg.2013.10.002

**Published:** 2013-11-07

**Authors:** Ge Zhang, Louis J. Muglia, Ranajit Chakraborty, Joshua M. Akey, Scott M. Williams

**Affiliations:** a Human Genetics Division, Cincinnati Children's Hospital Medical Center, Cincinnati, OH, USA; b Center for Prevention of Preterm Birth, Perinatal Institute, Cincinnati Children's Hospital Medical Center and March of Dimes Prematurity Research Center Ohio Collaborative, Cincinnati, OH, USA; c Center for Computational Genomics, Institute of Applied Genetics, University of North Texas Health Science Center, Fort Worth, TX, USA; d Department of Genome Sciences, University of Washington, Seattle, WA, USA; e Department of Genetics and Institute for Quantitative Biomedical Sciences, Geisel School of Medicine, Dartmouth College, Hanover, NH, USA

**Keywords:** Natural selection, Complex traits, Polygenic adaptation, Genome-wide association

## Abstract

It has recently been hypothesized that polygenic adaptation, resulting in modest allele frequency changes at many loci, could be a major mechanism behind the adaptation of complex phenotypes in human populations. Here we leverage the large number of variants that have been identified through genome-wide association (GWA) studies to comprehensively study signatures of natural selection on genetic variants associated with complex traits. Using population differentiation based methods, such as *F*_ST_ and phylogenetic branch length analyses, we systematically examined nearly 1300 SNPs associated with 38 complex phenotypes. Instead of detecting selection signatures at individual variants, we aimed to identify combined evidence of natural selection by aggregating signals across many trait associated SNPs. Our results have revealed some general features of polygenic selection on complex traits associated variants. First, natural selection acting on standing variants associated with complex traits is a common phenomenon. Second, characteristics of selection for different polygenic traits vary both temporarily and geographically. Third, some studied traits (e.g. height and urate level) could have been the primary targets of selection, as indicated by the significant correlation between the effect sizes and the estimated strength of selection in the trait associated variants; however, for most traits, the allele frequency changes in trait associated variants might have been driven by the selection on other correlated phenotypes. Fourth, the changes in allele frequencies as a result of selection can be highly stochastic, such that, polygenic adaptation may accelerate differentiation in allele frequencies among populations, but generally does not produce predictable directional changes. Fifth, multiple mechanisms (pleiotropy, hitchhiking, etc) may act together to govern the changes in allele frequencies of genetic variants associated with complex traits.

## Highlights

• Natural selection on variants affecting complex traits is a common phenomenon.
• Natural selection for polygenic traits varies both temporarily and geographically.
• Changes in allele frequencies as a result of selection can be highly stochastic.
• Multiple selection mechanisms act together on variants affecting complex traits.

## 1 Introduction

Natural selection is the driving force behind adaptive evolution (Barton, 2007). A comprehensive understanding of how natural selection affects human phenotypes could lead to insights into the evolutionary history and genetic architecture of such traits (Bamshad and Wooding, 2003, Biswas and Akey, 2006, Fu and Akey, 2013, Nielsen et al., 2007). Genes or genomic regions targeted by selection are typically functionally important and therefore identifying the regions under selection can provide important mechanistic information about how the polymorphism leads to phenotypic variation (Bamshad and Wooding, 2003, Biswas and Akey, 2006, Nielsen et al., 2007).

Many statistical tests have been developed to detect genetic signatures of selection acting through different evolutionary mechanisms and timescales (Oleksyk et al., 2010, Sabeti et al., 2006). These tests have been used to identify some genomic loci that harbor common alleles that underlie human adaptive traits (Fu and Akey, 2013, Grossman et al., 2013). Examples include *DARC* (Hamblin et al., 2002) in malaria resistance, *LCT* (Bersaglieri et al., 2004, Poulter et al., 2003) in lactose tolerance, *SLC24A5* (Lamason et al., 2005) in skin pigmentation, *EPAS1* (Yi et al., 2010) in high-altitude tolerance, and *EDAR* in adaptation to humid environment (Kamberov et al., 2013, Sabeti et al., 2007). Most of the above examples emphasize classic selective sweeps in which a new, strongly advantageous mutations increase rapidly in frequency, even to fixation, in the population. Strong positive selection for new variants can result in selective sweeps (Akey, 2009, Fu and Akey, 2013) that reduce the heterozygosity of nearby neutral polymorphisms and produces a strong genetic signature (i.e. a long haplotype with high frequency)(Sabeti et al., 2006), a process referred to as genetic hitch-hiking effect (Kaplan et al., 1989, Smith and Haigh, 1974). However, increasing empirical and theoretical studies (Hancock et al., 2010, Hermisson and Pennings, 2005, Hernandez et al., 2011, Przeworski et al., 2005) have suggested a role for “soft sweeps”, particularly adaptation by modest changes in allele frequencies at many standing variants — so called “polygenic adaptation”, could be the major mechanism behind most adaptive events in natural populations (Pritchard and Di Rienzo, 2010, Pritchard et al., 2010). Unlike strong selective sweep, polygenic adaptation depends on allele frequency changes at many loci and the selection signature at any one locus is generally weak and can therefore be difficult to detect by conventional methods (Hancock et al., 2010, Hermisson and Pennings, 2005, Przeworski et al., 2005).

Although being an old concept in classical quantitative genetics, polygenic adaptation has been largely neglected in human population genetics studies (Pritchard and Di Rienzo, 2010), probably due to our incomplete knowledge about the genetic architecture of complex human traits and the difficulties in detecting the weak selection signals at individual loci. Recently, genome-wide association (GWA) studies have revealed thousands of genetic variants associated with hundreds of complex human traits or diseases (http://www.genome.gov/gwastudies/) (Hindorff et al., 2009). The results from GWA studies not only confirmed the highly polygenic nature of most complex human traits (McCarthy et al., 2008, Stranger et al., 2011) but also enabled aggregating signals of selection across multiple trait associated variants, each of which might not stand out above the neutral background (Pritchard et al., 2010). For examples, Amato et al. (2011) found the mean *F*_ST_ of the 180 variants associated with human height to be significantly higher than the genomic background; Turchin et al. (2012) identified systematic allele frequency differences of height SNPs between Northern and Southern Europeans; Casto and Feldman (2011) demonstrated widespread selection on complex human traits through examination over 1300 GWAS SNPs; and Raj et al. (2013) observed significant enrichment of recent positive selection signatures among more than 500 inflammatory-disease susceptibility SNPs. However, there were also contradictory reports (Adeyemo and Rotimi, 2010, Ding and Kullo, 2011, Lohmueller et al., 2006, Myles et al., 2008) that did not detect unusually more differentiation of diseases associated variants between populations, which might suggest that positive selection does not have a strong effect on risk alleles in general.

In this study, we systematically examined nearly 1300 GWA SNPs associated with 38 complex phenotypes, ranging from quantitative physical or physiological traits to complex diseases. Instead of detecting selection signature at individual variants, we aimed to identify combined evidence of natural selection by aggregating signals across many GWA SNPs associated with a particular trait. Specifically, for a complex trait, we tested whether the GWA SNPs collectively showed accelerated differentiation among populations and tried to partition the observed differentiation into specific human lineage(s). To explore the potential fitness effect of the polygenic traits, we assessed the correlation between the effect sizes of the trait associated SNPs and the estimated strength of selection. We also investigated whether there is a significant trend in allele frequency changes that might favor directional shift in trait values or disease risks.

Our results demonstrate that, GWA SNPs of many complex traits are collectively more differentiated (in terms of allele frequencies) than the genome-wide average, supporting the conclusion that natural selection on variants associated with complex traits is a common phenomenon. In some traits, the accelerated changes in allele frequencies of GWA SNPs were mapped to specific human lineages, indicating that the selection forces on different polygenic traits vary both temporarily and geographically. In some traits with strong evidence of natural selection (e.g. height and urate level), we found significant correlation between the effect sizes and the estimated strength of selection in the trait associated SNPs, which might imply these traits could have been the primary targets of selection. But for most other traits, the accelerated allele frequency changes in trait associated SNPs might have been driven by the selection on other correlated phenotypes. Furthermore, we did not observe significant evidence of directional changes in allele frequencies among most of the traits examined in this study, which indicate polygenic selection might accelerate differentiation between populations without producing predictable directional changes in allele frequencies. Based on the inspection of individual SNPs showing significant signals of selection, we also found that multiple mechanisms (pleiotropy, hitchhiking, etc.) may act together to govern the changes in allele frequencies of complex trait associated SNPs.

## 2 Methods

### 2.1 Extraction of GWA SNPs of complex traits

We extracted GWA SNPs that are implicated in influencing complex human traits or diseases from the National Human Genome Research Institute (NHGRI) GWA catalog (http://www.genome.gov/gwastudies/). For a specific trait, we included autosomal GWA SNPs with p-value less than 5 × 10^− 8^ reported in studies with sample size larger than 1000. Since different studies might report different leading SNPs to index a significant locus, we selected the most significant one (with smallest reported p-value) from multiple reported GWA SNPs if they are physically adjacent to each other (< 250 kb). Our analyses of selection signatures (described below) required genotype data from the 1000 Genomes Project and ancestral state of the variants. Therefore, SNPs not presented in 1000 Genomes or whose ancestral allele cannot be determined were excluded. When possible, we also extracted the risk allele and the effect size estimates (beta or OR) of the GWA SNPs from the single study with the largest number of samples and reported significant SNPs.

### 2.2 1000 Genomes data

Genotype data from the 1000 Genomes Project (version 3 of the phase 1 integrated variant call set based on both low coverage and exome whole genome sequence data) were downloaded from ftp://ftp.1000genomes.ebi.ac.uk/vol1/ftp/release/20110521/. Of the 14 sampled 1000 Genomes populations, data from nine populations were used, including four European (EUR) populations (CEU, GBR, TSI, FIN), three East Asian (ASN) populations (CHB, CHS and JPT), and two African (AFR) populations (YRI and LWK). The CLM, MXL, PUR and IBS samples were excluded due to either high levels of admixture (CLM, MXL and PUR) or small sample size (IBS, N = 14). The ancestral alleles, constructed based on the 4-way EPO alignments (Paten et al., 2008a, Paten et al., 2008b) of human, chimp, orangutan and rhesus macaque sequences were extracted from the INFO section of the 1000 Genomes VCF files.

### 2.3 *F*_ST_ analysis

Unbiased estimates of *F*_ST_ were calculated according to Weir and Cockerham (1984). For each SNP under consideration, we calculated a global *F*_ST_ as well as all pairwise *F*_ST_ between the nine 1000 Genomes populations. We took the averages of selected pairwise *F*_ST_ to reflect the genetic distances between or within major continental populations. For examples: 1) the *F*_ST_ between African and Asian populations (AFR and ASN) was calculated by arithmetic mean of *F*_ST_ (YRI_CHB), *F*_ST_ (YRI_CHS), *F*_ST_ (YRI_JPT), *F*_ST_ (LWK_CHB), *F*_ST_ (LWK_CHS) and *F*_ST_ (LWK_JPT); 2) the *F*_ST_ within European population (EUR) was calculated by arithmetic mean of *F*_ST_ (CEU_GBR), *F*_ST_ (CEU_TSI), *F*_ST_ (CEU_FIN), *F*_ST_ (GBR_TSI), *F*_ST_ (GBR_FIN) and *F*_ST_ (TSI_FIN). The Weir and Cockerham estimation of *F*_ST_ can yield negative values, although do not have biological meaning, were kept to maintain the unbiased property of the estimator.

### 2.4 Branch length analysis

**Fig. 1:**
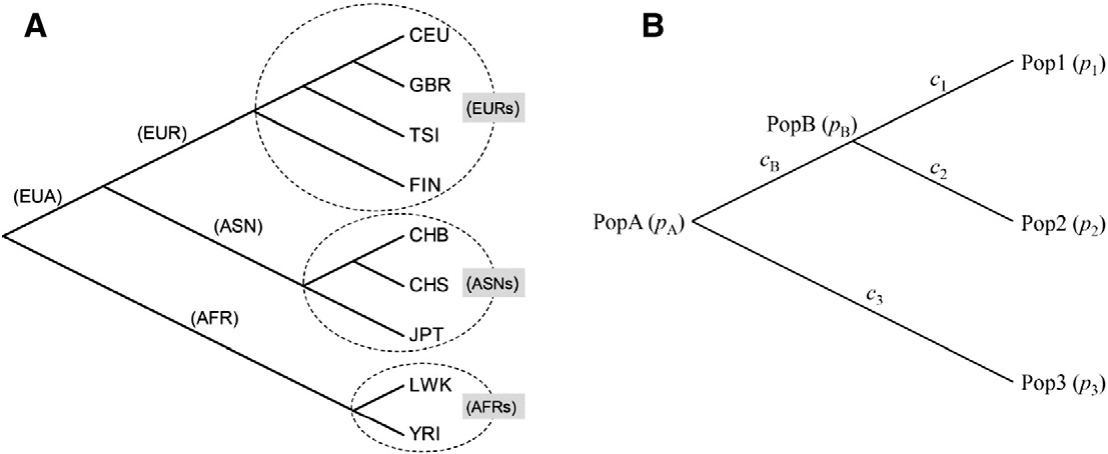
(A) Population tree (topology) of the nine 1000 Genomes populations. (B) schematic graph of branch length estimation.

To examine whether there are identifiable significant selection signals along specific human lineages, we estimated the branch lengths for the population tree of the nine studied populations (Fig. 1A). We first constructed the tree topology using the neighbor joining (NJ) method (Saitou and Nei, 1987) based on average pairwise *F*_ST_ estimated from 10,000 randomly selected 1000 Genomes SNPs. This topology was consistent with the one based on classic blood group and protein loci (Nei and Roychoudhury, 1993). Given this fixed topology, two methods were used to estimate the branch lengths based on the allele frequencies of each SNP: 1) the Ordinary Least Squares (OLS) method (Chakraborty, 1977, Rzhetsky and Nei, 1992); and 2) a maximum likelihood (ML) method based on the diffusion approximation (Kimura, 1962) of allele frequency changes. Specifically, the first method (OLS) estimates branch lengths by minimizing the squared errors between the observed genetic distances (i.e. pairwise *F*_ST_ between leaf nodes) and the distances over the tree (i.e. the sum of the branch lengths in the path between two leaf nodes). The second method (ML) was motivated by a hierarchical model of allele frequency changes among related populations (Nicholson et al., 2002), which assumes descendent populations diverge and evolve independently from an ancestral population (parental node) and the allele frequency in a descendent population is approximated by a normal distribution. As shown by a simple example (Fig. 1B), the allele frequency of PopB can be written as *p*_*B*_ ∼ *N*[*p*_*A*_, *c*_*B*_*p*_*A*_(1 − *p*_*A*_)], where *p*_*A*_ is the allele frequency in the ancestral population (PopA) and *c*_*B*_ is the branch length parameter relevant to the demographic history of PopB. Under pure drift setting, $c_{B}=\sum_{i=1}^{t_{B}}\frac{1}{2N_{i}}$, where *t*_*B*_ is the number of generations since PopB split from PopA and *N*_*i*_ is the effective population size of each generation. Conditional on the ancestral allele frequencies, the same model applies, independently, to all the non-root populations in a tree. Thus, the full likelihood of a population tree can be written as the product of the likelihoods of every non-root nodes:

$$L=\prod_{i}N\left[p_{i}^{*},c_{i}p_{i}\left(1-p_{i}\right)\right]$$

where *i* denotes each (non-root) population, *p*_*i*_ is the allele frequency of the population and *p*_*i*_^⁎^ is the allele frequency in the immediate ancestral population (parent node), and *c*_*i*_ is the branch length. The branch lengths (*c*_*i*_) and the allele frequencies in ancestral populations were then estimated by numerical maximization of the likelihood function. A detailed description of the method will be reported elsewhere. The branch lengths estimated by these two methods are similar and both can be interpreted as analogous to *F*_ST_ between a population and its hypothetical ancestor (parent node). To reduce the number of comparisons, we focused on the major splits of continental populations (i.e. AFR, EUA [Eurasia], ASN and EUR in Fig. 1A) and aggregated the terminal branches to reflect the average of within continental splits (indicated by EURs, ASNs and AFRs in Fig. 1A). The sum of branch lengths for the entire tree was also calculated.

### 2.5 Statistical test

For a particular complex trait, we inferred the aforementioned *F*_ST_ and the branch lengths (summarized in Table 1) from the allele frequency data of individual GWA SNPs. A permutation procedure was performed to test whether the inferred measures across all of the GWA SNPs associated with a particular trait collectively implies significant signature of selection. 5000 permutation sets of SNPs were drawn at random from the 1000 Genomes data and matched to the GWA SNPs on a one-by-one basis by having similar ancestral allele frequency in the European 1000 Genome samples. The distributions and mean values of the estimated *F*_ST_ and the branch lengths obtained from the GWA SNPs were then compared to the permutation sets to calculate empirical p-values. Since we were interested in detecting signatures of selection that lead to accelerated population differentiation, one-tailed p-values for increased *F*_ST_ and branch lengths were used. Similar empirical p-values were also calculated for individual GWA SNPs to reflect the significance of selection on individual GWA SNPs.

Multiple (38) complex traits and a large number (nearly 1300) of GWA SNPs associated with these traits were examined in this study. However, we did not apply stringent corrections to account for multiple testing, because we assume natural selection is a common phenomenon and a multiple testing correction based on family-wise error rate (i.e. Bonferroni correction) would be too stringent. Instead, we arbitrarily set two levels (significant p-value < 0.01 and marginally significant p-value < 0.05) to rank the significance of evidence for selection.

### 2.6 Correlation between effect sizes and strength of selection

We also examined whether the strength of selection signatures are correlated with the reported effect sizes of the GWA SNPs. In classical quantitative genetics, strength of natural selection with respect to a genotype (or a phenotype) is measured by fitness (Falconer and Mackay, 1996). Differences in fitness can be used to predict the allele frequency change over generations. However, despite its core importance, it is almost impossible to measure small fitness differences among genotypes in natural populations (Orr, 2009). In this current study, we used estimated *F*_ST_ (global) and branch length (total) as surrogates to reflect the strength of directional selection. It can be shown that, when a directional selection with no dominance is assumed, the estimated *F*_ST_ and branch length should have an approximately linear relationship with the expected allele frequency change *Δq* = *sq*(1 − *q*)/2, where *q* is the allele frequency before selection, *s* is the selection coefficient, and the fitness for each genotype AA, Aa and aa are 1, 1 − *s*/2, and 1 − *s*, respectively. Following this logic we tested correlation between the estimated strength of selection signatures (i.e. *F*_ST_ or branch length) and the allele frequency adjusted effect sizes *βq*(1 − *q*), where *β* is the reported beta values or log transformed ORs and *q* is the allele frequency in European populations. The statistical significance of the correlation was accessed by both parametric (Pearson correlation) and non-parametric (Kendall's *tau*) methods.

### 2.7 Tests for directional changes in allele frequencies

Suppose natural selection favors directional change (e.g. increasing) in trait value of a complex phenotype (or risk to a complex disease) — under this premise, we should anticipate observing more derived alleles that increase the trait value than derived alleles that decrease the trait value and the frequencies of derived alleles with increasing effects should be higher than the derived alleles with decreasing effects or vice versa. Therefore, we grouped the reported GWA SNPs according to the effect direction of the derived alleles (i.e. positive or negative) and tested whether the counts and the derived allele frequencies of the two groups of GWA SNPs are significantly different as overall evidence for directional selection. A two-tailed binomial test was conducted to compare the counts of derived alleles and a two-sample Wilcoxon test was used to compare the frequency distributions of the derived alleles with positive or negative effects.

## 3 Results

### 3.1 Extraction of complex traits and GWA SNPs

We compiled a list of complex traits and their associated GWA SNPs from the NHGRI GWA catalog (http://www.genome.gov/admin/gwascatalog.txt, downloaded on May 13, 2013). This version of GWA catalog includes 12,430 GWA records on 826 traits from 1770 published studies. Since our study aimed at identifying collective selection signatures on standing genetic variants of complex traits by aggregating evidence from multiple GWA SNPs, we included 38 complex traits/diseases with more than 15 index GWA SNPs that passed our selection criteria (i.e. autosomal GWA SNPs with p-value < 5 × 10^− 8^). Because of the implications for natural selection upon psychiatric disorders genes (Keller and Miller, 2006), we included Schizophrenia and Bipolar disorder by lowering the p-value < 5 × 10^− 6^. The selected traits and the counts of GWA SNPs were listed in Table 2. The 38 traits can be grossly grouped into 7 categories: 1) quantitative physical traits (i.e. height, BMI and blood pressures); 2) quantitative physiological traits; 3) inflammatory or autoimmune disorders; 4) common complex disorders; 5) mental disorders 6) cancers; and 7) a miscellaneous category related to menstruation. In total, 1458 significant GWA associations of 1296 distinct SNPs (a SNP could associate with multiple traits) were extracted, and effect size information was available for 1082 GWA SNP associations.

In the following, we first detail our results from height GWA SNPs to illustrate the logical organization of our multifaceted analyses. The results from other traits were briefly summarized in Table 3, Table 4 (detailed results organized in similar way to height can be found on our web site: http://tgx.uc.edu/zg/polygen_selection).

### 3.2 Height: An example

#### 3.2.1 GWA SNPs

From the GWA catalog we exacted 178 height SNPs. Effect size and direction of 167 SNPs reported by (Lango Allen et al., 2010) were available.

#### 3.2.2 *F*_ST_ metrics

**Fig. 2:**
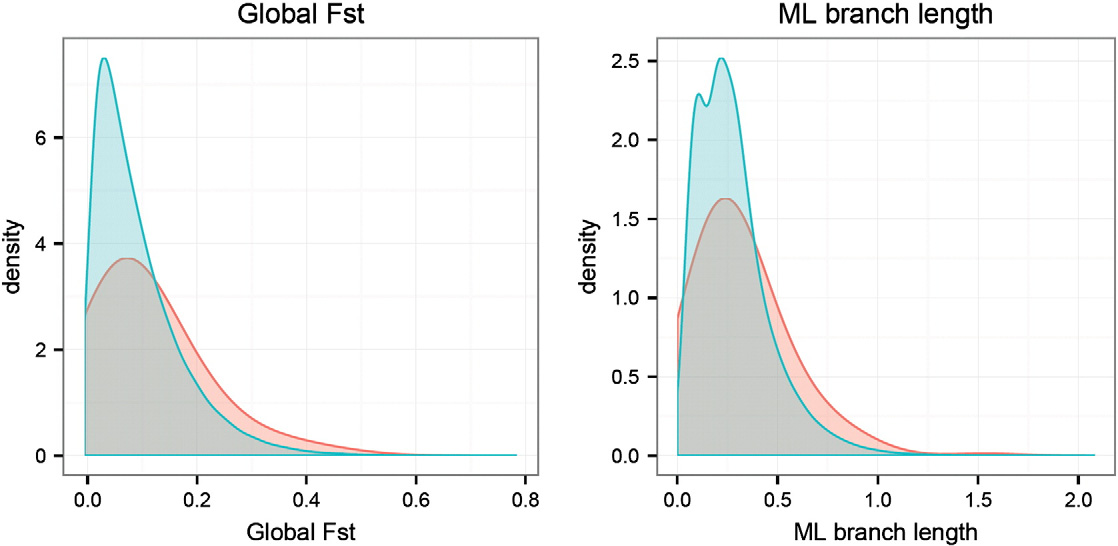
Distributions of the global *F*_ST_ (left) and the total branch length (right) estimated from the height GWA SNPs (red) in contrast to genome background (blue).

**Fig. 3:**
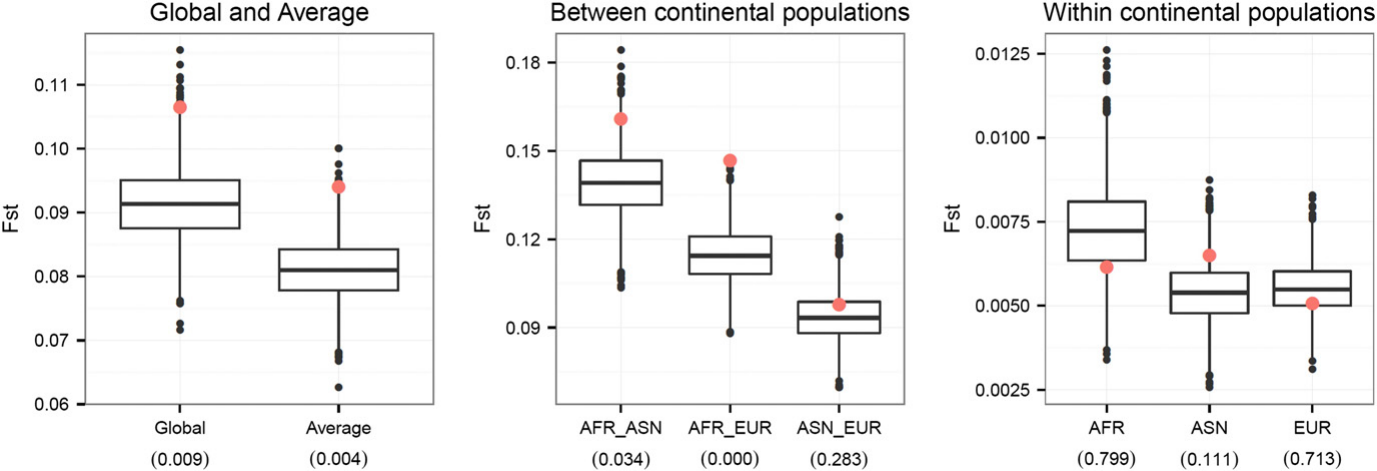
Box plots of mean *F*_ST_ estimated from the randomly selected permutation sets of SNPs (genome background) and the mean *F*_ST_ estimated from the height GWA SNPs (red dots). The numbers in parentheses are the empirical p-values.

Our first analysis of selection signatures was based on the *F*_ST_ metric. Compared to the “null” SNP distributions derived from the 5000 permutation sets matched by ancestral allele frequencies, the distribution of the overall *F*_ST_ of the height SNPs clearly had an increased mean and variance (Fig. 2A). The empirical p-value of the average of the overall *F*_ST_ across the 178 height SNPs was 0.0088, indicating increased differentiation of height SNPs among populations compared to the genomic background (Fig. 3). The most significant differentiation was observed between African and European populations, as indicated by the high mean *F*_ST_ (AFR_EUR) between these two continental populations (p-value = 0.0004). The differentiation between African and Asian populations (AFR_ASN) was only marginally significant (p-value = 0.034) and no significantly increased differentiation was observed between Asian and European populations (ASN_EUR) or within the three continental populations.

#### 3.2.3 Branch length analysis

**Fig. 4:**
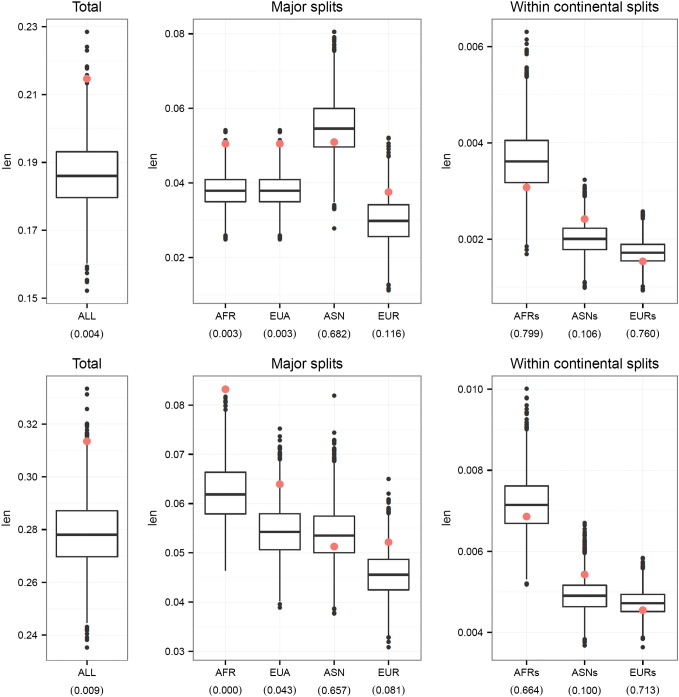
Box plots of mean branch lengths estimated by either OLS (top) or ML (bottom) from the randomly selected permutation sets of SNPs (genome background) and the mean branch lengths estimated from the height GWA SNPs (red dots). The numbers in parentheses are the empirical p-values.

Our second analysis estimated phylogenetic branch lengths using OLS and ML. In general, the two methods gave similar results, though the ML method tended to report more significant p-values (Fig. 4). Similar to the distribution of global *F*_ST_, the total branch lengths estimated from the height SNPs were significantly longer than the “null” permutation SNPs (Fig. 2B). The most significant elongated branch lengths were observed along the African (AFR) and Eurasia (EUA) splits, which might suggest most of the differentiation in allele frequencies between African and European or Asian populations was accumulated during this period.

#### 3.2.4 Correlation between effect sizes and strength of selection

**Fig. 5:**
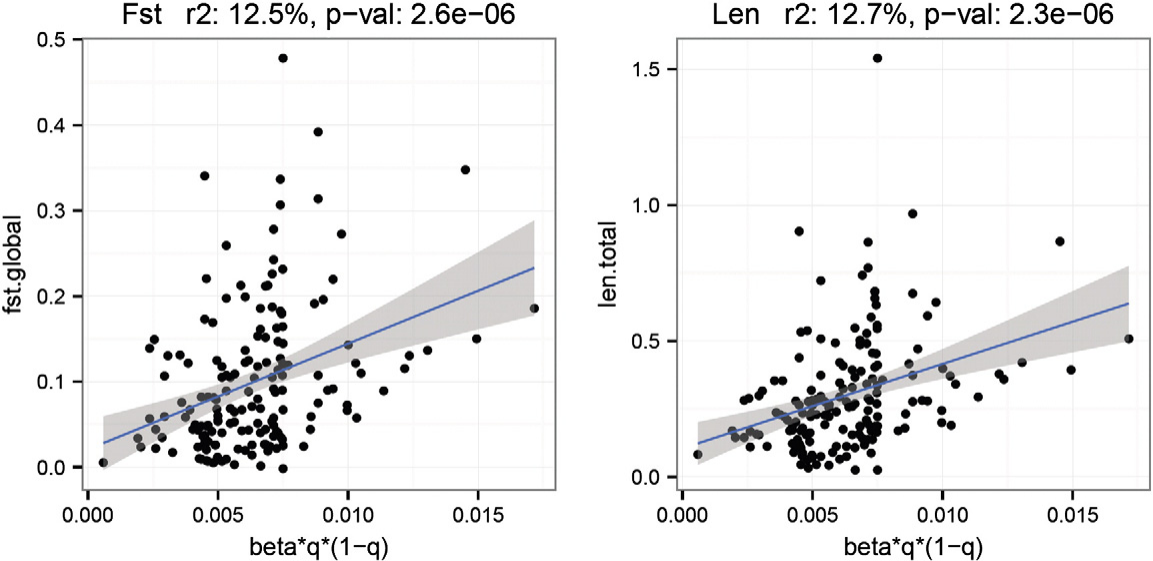
Dot plots between the allele frequency adjusted effect size and the global *F*_ST_ (left) or total branch length (right) of the height GWA SNPs.

We identified significant correlation between the allele frequency adjusted effect sizes *βq*(1 − *q*) and the strength of selection inferred by overall *F*_ST_ or the total branch lengths. Based on linear regression (Fig. 5), the allele frequency adjusted effect sizes explained approximately 12.5% (p-value = 2.6 × 10^− 6^) and 12.7% (p-value = 2.3 × 10^− 6^) of the total observed variances in the overall *F*_ST_ and the total branch lengths respectively. The rank correlation test (Kendall's *tau*) reported even more significant p-values (8.7 × 10^− 7^ and 6.0 × 10^− 8^).

#### 3.2.5 Tests for directional changes

**Fig. 6:**
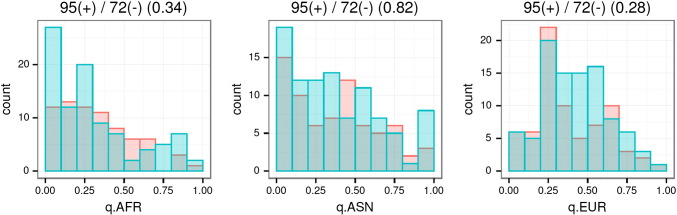
Frequency distributions of derived alleles (blue: alleles with positive effect; red: alleles with negative effect) of the height GWA SNPs in the three continental populations. The numbers of derived alleles with positive (+) or negative (−) effect and the Wilcoxon test p-values (in parentheses) are shown above the figures.

Among the 167 SNPs with effect size and direction information, derived alleles of 95 SNPs increase height and 72 SNPs decrease height (binomial p-value = 0.088). Based on the 1000 Genomes data, we did not observe significant differences of the derived allele frequencies between these two sets of SNPs among the three major continental populations (Fig. 6). Our ML estimation of branch lengths also enabled estimation of allele frequencies in ancestral populations, and again, we did not detect evidence for preference of directional changes of allele frequencies of the height SNPs between ancestral and current populations (Online supplementary material).

#### 3.2.6 Individual SNPs

Four height SNPs showed putative signal of selection, i.e. significantly large (p-value < 0.01) overall *F*_ST_ or total branch length (ML). The most significant one was rs1490384 on chromosome 6q22 near *CENPW* gene. This SNP was also reported to be associated with tooth eruption (Fatemifar et al., 2013). Another SNP was rs3791675 in the *EREMP1* gene, which was reported to be associated with growth rate in children (Paternoster et al., 2011). The other two significant SNPs (rs9835332 and rs143384) are located in *FAM208A* (also known as *RAP140*) and *GDF5* gene, respectively. Both genes showed evidence of recent selection, particularly the *GDF5* gene, which was highly significant in iHS analysis and had been suggested to be under positive selection in East Asians (Wu et al., 2012).

### 3.3 Selection signatures of other complex traits

#### 3.3.1 BMI and blood pressure

In addition to body height, we examined three other physical quantitative traits (BMI, systolic and diastolic blood pressures). Overall, we did not detect major signatures of selection from the collective analyses of the GWA SNPs of these traits, although the blood pressure SNPs showed accelerated differentiation among the four European populations (*F*_ST_ (EUR) p-value = 0.0022 and 0.0054 respectively, for SBP and DBP). At the individual SNP level, none of the BMI SNPs had empirical p-value (for overall *F*_ST_ or total branch length) less than 0.01. A non-synonymous SNP (rs3184504 in *SH2B3* gene) associated with blood pressure showed significant differentiation between European and non-European populations (*F*_ST_ p-value = 0.0042; branch length p-value = 0.0088). The derived allele (T) was rare in African and Asian populations (q = 0.03 and 0.01) but had high minor allele frequency (q = 0.47) in the European population. Another similar but less significant example was an intronic SNP (rs1378942) in *CSK* gene, whose derived allele frequency (A) was high in EUR but low in ASN and rare in AFR.

#### 3.3.2 Lipids traits

Among the four lipid traits, the most significant selection signal was observed in triglycerides (TG) SNPs. The overall *F*_ST_ and total branch length of TG SNPs were significantly higher than genome-wide average with empirical p-value = 0.0094 and 0.0016, respectively. Even more significant differentiation was observed between African and Asian populations (*F*_ST_ (AFR_ASN) p-value = 0.0042) and within Asian populations (*F*_ST_ (ASN) p-value = 0.0032). Branch length analysis suggested accelerated differentiation along the African (p-value = 0.0072) and Eurasia (p-value = 0.030) split and Asian split (p-value = 0.037), but not the European split (p-value = 0.866). Several distinct TG SNPs (rs7819412 of *XKR6* gene and rs11649653 near *CTF1*) showed high derived allele frequency in Asian but intermediate frequency in European and low frequency in African populations. The HDL SNPs also showed some level of increased differentiation among the Asian populations, but did not reach nominal significance in empirical tests. The individual significant HDL SNPs (e.g. rs386000, rs3136441 and rs7255436) shared similar but less significant pattern of elevated derived allele frequencies in Asian populations. In contrast, the individual significant SNPs (e.g. rs7570971 in *RAB2GAP1* gene and rs11065987) found in total cholesterol or LDL had more distinct higher derived allele frequencies in Europeans but low frequencies in both Africans and Asians. The rs11065987 near the *BRAP* and *ATXN2* gene was associated with both LDL and TC and has been reported to be a risk SNP of Tetralogy of Fallot (Cordell et al., 2013). In addition, increased differentiation was observed in TC SNPs in European populations (*F*_ST_ (EUR) p-value = 0.003).

#### 3.3.3 Other metabolites and physiological traits

In the study of several other metabolites and physiological traits, we detected significant overall selection signature in GWA SNPs associated with serum urate level (*F*_ST_ (ALL) p-value = 0.008). Nominally significant differentiation was observed between European and African/Asian populations and within European populations particularly along path to the TSI population (Tuscans from Italy). The identified strength of selection was also significantly correlated with the reported effect sizes of the urate SNPs. Individually significant SNPs include rs675209 near the *RREB1* gene and rs653178 in the *ATXN2* gene. The later SNP has been reported as associated with blood pressure (Newton-Cheh et al., 2009, Wain et al., 2011), celiac disease (Dubois et al., 2010, Hunt et al., 2008) and chronic kidney disease (Kottgen et al., 2010).

The 53 GWA SNPs associated with platelet counts did not show significant signatures of selection, but one of the most significantly associated SNP — rs3184504 in *SH2B3* showed a significant difference in derived allele frequency between European and non-European populations. This SNP was also significantly associated with other blood cell traits (Gudbjartsson et al., 2009, Tin et al., 2013, van der Harst et al., 2012), blood pressure (International Consortium for Blood Pressure Genome-Wide Association, S., et al., 2011, Levy et al., 2009) and cardiovascular risks (Gudbjartsson et al., 2009, International Consortium for Blood Pressure Genome-Wide Association, S., et al., 2011, Schunkert et al., 2011), multiple autoimmune disorders, such as type 1 diabetes (Barrett et al., 2009, Plagnol et al., 2011), rheumatoid arthritis (Stahl et al., 2010), celiac disease (Izzo et al., 2011), multiple sclerosis (Alcina et al., 2010), hypothyroidism (Eriksson et al., 2012), etc. This variant had been reported in previous natural selection studies (Ding and Kullo, 2011, Pickrell et al., 2009, Sabeti et al., 2006, Zhernakova et al., 2010).

We did not observe significant selection signature from the collective analyses of 18 C-reactive protein (CRP) SNPs and none of the SNPs individually showed significant differentiation among the studied populations. Similar negative results were obtained from the analysis of 25 GWA SNPs of liver enzyme levels (gamma-glutamyl transferase, GGT) or the 16 GWA SNPs associated with adiponectin levels. However, the 14 adiponectin SNPs with effect information showed significantly unbalanced effects — the derived alleles of 13 SNPs have negative effects on adiponectin levels. The allele frequency differences of these SNPs between African and European population seemed to be small (*F*_ST_ (AFR_EUR) p-value = 0.992). The analysis of GWA SNPs associated the two electrophysiology traits (ventricular conduction and QT interval) yielded no significant results. Negative results were also observed in the 27 SNPs associated with corneal thickness, although significant differentiation among European populations was observed.

#### 3.3.4 Inflammatory and autoimmune disorders

Eleven inflammatory and autoimmune disorders were examined, including inflammatory bowel disease (IBD) and its subforms, (i.e. Crohn's disease, ulcerative colitis (UC)), celiac disease (CD), systemic lupus erythematosus (SLE), rheumatoid arthritis (RA), psoriasis, type 1 diabetes (T1D), multiple sclerosis (MS), vitiligo and primary biliary cirrhosis (PBC).

Among the inflammatory and autoimmune disorders the most significant collective selection signatures were obtained in GWA SNPs associated with SLE and vitiligo. The 29 SLE SNPs were significant for both global *F*_ST_ (p-value = 0.008) and branch length analyses (p-value = 0.0072). Most of the differentiation in SLE SNPs allele frequencies was driven by differences between African and European populations (*F*_ST_ (AFR_EUR), p-value = 0.0028) or the Eurasia split in branch length analysis (p-value = 0.001). A risk SNP (rs6705628) identified in Asian samples (Yang et al., 2013) had a low derived allele frequency in European (q = 0.01) but high frequency in Africans (q = 0.36) and Asians (q = 0.19).

Significant selection signatures were detected in the collective analysis of the 25 GWA SNPs associated vitiligo (global *F*_ST_ p-value = 0.0262 and the total branch length p-value = 0.0086). Between populations *F*_ST_ and branch length analysis demonstrated these significant signals were mainly driven by accelerated allele frequency changes along the European ancestry (branch length, EUR p-value = 0.0046) or the subdivision of different European populations (branch length, EURs p-value = 0.0028). Individual SNP analyses revealed significant selection signals in a SNP (rs4766578) in the *ATXN2* gene and a 3′UTR SNP (rs1129038) in *HERC2* gene. The *ATXN2* (and the nearby *SH2B3* gene) appeared as a candidate region that contains multiple GWA SNPs showing significant selection signature. The second SNP (rs1129038) is part of a haplotype spanning the *OCA2* and the *HERC2* gene, significantly associated with hair and eye colors (Candille et al., 2012, Donnelly et al., 2012, Eiberg et al., 2008, Kayser et al., 2008).

The collective analyses of the 108 IBD GWA SNPs as well as 95 Crohn's disease and 57 UC GWA SNPs did not show significant signatures of selection, although marginally elevated *F*_ST_ were observed between or within Asian and European populations in SNPs associated with Crohn's disease or UC. Individual SNP analysis showed that the derived allele frequencies of several risk SNPs (e.g. rs12103, rs670523, rs2472649, rs7517810 and rs11150589) were elevated in European or Asian populations, but the effects of the derived alleles of these SNPs pointed in both directions (either increasing or decreasing the risks), inconsistent with a common pattern of directional selection among populations.

The analysis of 27 CD GWA SNPs also did not reveal any major selection signatures, except for a marginally significant difference between African and European populations (*F*_ST_ (AFR_EUR), p-value = 0.035). Two CD SNPs were significant in individual SNP analysis: rs13003464 in *PUS10* and rs653178 in *ATXN2*. The first one was also associated with Crohn's disease (Kenny et al., 2012) and the second one had appeared before in our analysis of uric acid GWA SNPs. Rs653178 was also in close linkage disequilibrium with rs3184504, which was a significant SNP associated with platelet counts and many other disorders (see above).

The T1D GWA SNPs showed marginal significance in overall *F*_ST_ (p-value = 0.046) and branch length analysis (p-value = 0.033), but more significant differentiation was observed between Asian and European populations. Branch length analysis also indicated accelerated differentiation of T1D SNPs after the European population split from Eurasia (p-value = 0.025). Two T1D SNPs (rs9388489 and rs3184504) were significant in individual SNP analysis. The first one (rs9388489) is near rs1490384 — a height SNP with sign of selection; and the second one (rs3184504 in *SH2B3*) is associated with multiple complex traits.

The analysis of 48 MS SNPs showed no significant finding except for a marginally increased difference within the Asian populations (*F*_ST_ (ASN), p-value = 0.021). The individual SNP with most significant selection signal (rs7923837 in *HHEX*) was also associated with Type 2 diabetes (Cai et al., 2011, Staiger et al., 2007), particularly significant in Asian populations (Horikoshi et al., 2007).

Collective analysis of GWA SNPs associated with RA, psoriasis and PBC did not show significant collective signature of selection. A PBC associated SNP (rs968451) near PDGFB were in close linkage disequilibrium with rs2413583, which is associated with IBD and Crohn's disease.

#### 3.3.5 Other complex disorders

The analysis of 49 type 2 diabetes (T2D) suggested marginally increased differentiation of T2D SNPs among populations (*F*_ST_ (ALL) p-value = 0.0354), which was likely attributed to the Eurasia split from Africa. The individual T2D SNP showing most significant selection signal was rs8042680 in *PRC1* gene, which has a high derived allele (protective) frequency in European but is rare in African and absent in Asian.

The analysis of 35 coronary heart disease (CHD) did not show overall increased differentiation among populations (overall *F*_ST_ p-value = 0.133), except for a marginal increase between African and European populations (*F*_ST_ (AFR_EUR), p-value = 0.034). The individual CHD SNP showing most significant selection signal was rs599839 in *PSRC1* gene, which was also significantly associated with LDL (Sandhu et al., 2008, Willer et al., 2008).

In the study of two mental disorders, we observed strong selection signals in the 49 schizophrenia SNPs (global *F*_ST_ p-value = 0.016 or total branch length p-value = 0.007). The most significant allele frequency differences of schizophrenia SNPs were observed between African and non-African populations (*F*_ST_ (AFR_ASN), p-value = 0.0048, *F*_ST_ (AFR_EUR), p-value = 0.0026) and were mostly attributed to the African (AFR p-value = 0.0004) and Eurasia (EUA p-value = 0.026) splits in branch length analysis. The 42 bipolar SNPs did not show overall increased allele frequency differences among populations, however, the differences among European populations was significant (*F*_ST_ (EUR), p-value = 0.011).

#### 3.3.6 Cancers

We examined GWA SNPs associated with three different types of cancers (breast, prostate and colorectal cancers). The most significant collective evidence of population differentiations was observed in the 34 SNPs associated with prostate cancer (global *F*_ST_ p-value = 0.017 or total branch length p-value = 0.01). Much of the differentiation was mapped to the African lineage in the ML branch length analysis (p-val (AFR) = 0.0002). Two significant SNPs (rs1465618, rs103294) are located in *THADA* and near *LILRA3* gene, respectively. Multiple SNPs (rs7590268, rs6732426, rs13429458, rs17030845, rs12478601, rs7578597 and rs10495903) in the *THADA* gene have been reported to be associated with various complex traits or diseases: cleft palate (Ludwig et al., 2012, Mangold et al., 2010), hair morphology (Medland et al., 2009), polycystic ovary syndrome (Chen et al., 2011, Shi et al., 2012), platelet counts (Gieger et al., 2011), type 2 diabetes (Zeggini et al., 2008), IBD and Crohn's disease (Franke et al., 2010, Jostins et al., 2012). This gene has also been reported as a gene under selection (Ding and Kullo, 2011, Klimentidis et al., 2011, Pickrell et al., 2009).

The GWA SNPs associated with breast or colorectal cancers did not show an overall significant signature of selection. But there was a sign of elevated differentiation of colorectal cancer SNPs among Asian populations (*F*_ST_ (ASN) p-value = 0.0006). The significant colorectal cancer SNP (rs4925386 in *LAMA5* gene) has higher derived allele frequency in Africans, but relatively low frequencies in Asians and Europeans. iHS scores were also positive, suggesting selection on ancestral alleles, which was a rare example of this kind in our list of significant GWA SNPs under selection (Table 4).

#### 3.3.7 Menstruation related traits

We also examined two menstruation traits: age of onset of menarche and menopause. The evolutionary significance of menstruation has been hypothesized (Emera et al., 2012, Renfree, 2012, Stearns, 2012). However, our analysis of GWA SNPs associated with these two traits did not reveal any significant sign of selection, except for a single variant, rs1361108, associated with age of menarche that showed significant allele frequency differences between populations. This variant is in close LD with rs9388489 and rs1490384, associated with T1D and height, respectively.

## 4 Discussion

The results of GWA studies suggest that most complex human traits are highly polygenic (McCarthy et al., 2008, Stranger et al., 2011). As a result, the effects of polygenic adaptation on genetic variants associated with complex traits will be generally modest and will spread across many loci (Hancock et al., 2010, Pritchard and Di Rienzo, 2010, Pritchard et al., 2010). Therefore, in this current study, we aimed to identify combined evidence for natural selection of complex traits by aggregating selection signals across multiple GWA SNPs. Specifically, for a complex trait, we examined 1) whether the GWA SNPs collectively show accelerated differentiation among populations; 2) in which human lineage(s) the allele frequencies underwent significant changes; 3) whether the strength of selection is correlated with the effect size of the genetic variant; and 4) whether the changes in allele frequencies are directional or not. Through analyses of nearly 1300 GWA SNPs associated with 38 complex traits, we revealed some general features of natural selection on complex traits associated variants.

### 4.1 Selection signatures of complex traits

Among the 38 complex traits/diseases examined in this study, we observed evidence for natural selection in many traits based on the collective analyses of trait associated SNPs. The most notable examples include: body height, serum urate levels; triglycerides (TG); multiple autoimmune disorders, particularly SLE and vitiligo; schizophrenia; and less significantly, type 2 diabetes (T2D) and prostate cancer. For those traits that did not reach our predefined significance level (p-value < 0.01), the distribution of the global *F*_ST_ or total branch length estimated from the GWA SNPs generally had higher mean and larger variance than the random SNPs, supporting the hypothesis that natural selection on variants associated with complex traits is a common phenomenon (Casto and Feldman, 2011, Pritchard and Di Rienzo, 2010, Pritchard et al., 2010).

The most significant example of combined signature of selection as indicated by elevated mean global *F*_ST_ and total branch length was observed in the 178 height association SNPs. This result generally agrees with two recent studies (Amato et al., 2011, Turchin et al., 2012). Amato et al. (2011) found a higher mean *F*_ST_ of the height SNPs based on the HapMap data and Turchin et al. (2012) demonstrated systematic allele frequency differences of height SNPs between Northern and Southern Europeans, both indicating that natural selection may be acting on the height associated SNPs. Our branch length analysis partitioned the major differentiations to the early splits between African and Eurasia lineages instead of more recent splits, suggesting the selection force on these height SNPs is more of a factor early in human migration. Anthropological evidences indicated there was a body size decrease in humans 50,000 years ago possibly associated with technological improvements in food production and climate change (Ruff, 2002).

Our second most significant example with collective evidence for selection was urate levels — the mean global *F*_ST_ and total branch length estimated from the 29 urate SNPs were significantly higher than permutation sets. Unlike the height SNPs, significant differentiations in allele frequencies were observed between European and Asian populations and within European populations particularly along path to the TSI population. Uric acid (UA) is the end product of purine metabolism and humans have higher UA levels than other mammals due to the loss of uricase activity. Despite its harmful effects, the evolutionary benefits of uric acid as an antioxidant and neuroprotector have been hypothesized (Alvarez-Lario and Macarron-Vicente, 2010, Johnson et al., 2005), and the selection upon urate SNPs may be associated with dietary changes (e.g. the intake of red meat and alcohol consumption).

Among the four lipid traits, we observed significant evidence for selection in the 33 SNPs associated with TG level. Based on our branch length analysis, the selection signals dispersed across multiple population lineages except for European populations. TG is the main way for energy storage (Hegele, 2009) and has an essential role in human evolution. The selection on TG associated variants may be relevant to physiological adaptions to nutritional stress that modern humans have experienced in evolution history (Nakayama et al., 2011). As compared to other lipids, TG may be more associated with environmental changes (i.e., diet) and may therefore play a role in changing diets over human evolution.

We detected selection signatures in multiple inflammatory and autoimmune disorders. But compared to other physical or physiological traits and complex diseases examined in this study, these autoimmune disorders did not show enrichment or stronger signatures of selection as suggested by recent studies (Barreiro and Quintana-Murci, 2010, Raj et al., 2013). Among the eleven autoimmune disorders examined in this study, the SNPs associated with SLE and vitiligo showed the strongest evidence of allele frequency differentiation.

Evolutionary hypotheses such as the thrifty-genotype hypothesis (Neel, 1962) and the ancestral-allele susceptibility model (Di Rienzo and Hudson, 2005) have been proposed to explain the epidemiology of complex diseases in the context of evolution. All these models implicate genetic variants that have undergone positive selection during historical periods but might be maladaptive in present day environment and give rise to disease phenotypes (Biswas and Akey, 2006). Indeed, we observed marginally significant (p-value < 0.05) signatures of selection in the analyses of GWA SNPs associated with type 2 diabetes (T2D) and coronary heart disease (CHD), but the there are no consistent patterns of selection that provide conclusive support to these evolutionary hypotheses.

Severe mental disorders impart clear decreases in fitness and as such, why susceptible alleles to mental diseases are still maintained in population is a paradox (Keller and Miller, 2006). In the analyses of GWA SNPs associated with two major types of mental disorders, we found signature of selection among the SNPs associated with schizophrenia but not in the bipolar GWA SNPs, which might suggest different evolutionary scenarios of these two mental disorders.

We also observed significant signatures of selection in the SNPs associated with prostate cancer and the accelerated differentiation was mainly mapped to the African lineage. The SNPs associated with colorectal cancer, although did not show more differentiation globally, were significantly more differentiated among Asians.

In summary, by aggregating evidence of population differentiation across many GWA SNPs associated with a complex trait, we detected significant combined signatures of selection in variants associated with many complex traits and diseases. These results were consistent with (Adeyemo and Rotimi, 2010, Amato et al., 2009, Ding and Kullo, 2011, Kullo and Ding, 2007), which also showed genetic variants associated with complex human diseases show wide variation across multiple populations. The power of our method varies between different traits due to the different numbers of identified GWA SNPs. In addition, we used arithmetic mean to summarize the selection measures (i.e. *F*_ST_ or branch length), which would average out real selection signals when there are both directional (increase in *F*_ST_) and balancing (decrease in *F*_ST_) selection in the same set of SNPs. Although compared to directional selection, balancing selection might be less common (Akey et al., 2002) and usually has a less significant footprint (Beaumont and Balding, 2004). Indeed, we observed SNPs with unusually small differentiation (suggesting balancing selection), such as a SNP associated with IBD (rs12942547 in *STAT3* gene, p-value = 0.998 in our one directional test for increased differentiation). The same SNP has been reported to be associated with both IBD and MS but with opposite effect (Cagliani et al., 2011, Jostins et al., 2012). Recently, antagonistic pleiotropy was suggested to drive the balancing selection of *OLR1* gene in certain human populations (Predazzi et al., 2013).

### 4.2 Selection signature along specific lineages of human populations

In addition to the *F*_ST_ analyses, we tried to partition the observed allele frequency differences among different populations into particular lineage(s) of human evolution by branch length analysis. Similar methods have been proposed before (Pickrell and Pritchard, 2012, Shriver et al., 2004). Based on a fixed topology (Fig. 1A) supported by both 1000 Genomes data and previous population genetics studies, we used two different analytical methods to obtain the branch length estimates: the OLS method based on pairwise *F*_ST_ and an ML method which directly models allele frequency changes among related populations. The results generated by these two methods were in close agreement, except for some variations in the estimation of terminal branch lengths. The estimated branch lengths can be thought of as an analogous to *F*_ST_, between a population and its hypothetical ancestor. Using this approach, we were able to map selection signals along specific population evolutionary lineages.

Of the complex traits/diseases with significant signatures of selection, the patterns of branch lengths and the associated empirical significances showed by the GWA SNPs vary between traits, indicating different evolutionary history of different traits, or in other words recent adaption of different complex traits might be associated with temporarily and geographically specific environmental challenges. Furthermore, we did not observe pervasive significant branch length changes spreading over the population tree, indicating the selection forces on a polygenic trait vary both temporarily and geographically in human evolutionary history. For example, the SNPs associated with height, SLE, schizophrenia and prostate cancer showed most significant differentiations between African and European populations and the accelerated changes in allele frequencies were mapped deeply to African-Eurasia splits. But the differentiation in allele frequencies of SNPs associated with urate levels and vitiligo seems having a relatively recent origin between the European and the Asian split. There were also examples whose associated SNPs expressed significant differentiation within continental populations, such as SNPs associated with blood pressure, total cholesterol, corneal structure and vitiligo were highly differentiated among European populations. But SNPs associated with triglycerides and colorectal cancer were increasingly differentiated among Asian populations.

Because of the highly stochastic nature of allele frequency changes among populations, substantial uncertainty might exist in our branch length estimation, particularly considering the number of variables (number of branches) that need to be estimated is far more than the number of observations (number of populations). Also, the population tree model (Fig. 1) behind our branch analyses is an oversimplification of human population history, which assumes absolute geographical isolation and ignores gene flow (Pickrell and Pritchard, 2012). In addition, individual SNPs associated with a particular trait might undergo different evolutionary history and cannot be represented accurately by an average pattern across multiple SNPs.

### 4.3 Correlation between strength of selection and effect size

In several of the complex traits examined in this study, we detected significant correlations between the reported effect sizes (allele frequency adjusted) of the SNPs and the estimated *F*_ST_ (global) or branch length (total). As the *F*_ST_ and branch length capture cumulative genetic response to selection over generations, these measures were used here as surrogate measures of the “strength of selection”. The most significant example again was height, the allele frequency adjusted effect size *βq*(1 − *q*) was highly correlated with the estimated global *F*_ST_ (p-value = 2.6 × 10^− 6^) or total branch length (p-value = 2.3 × 10^− 6^) and it can explain a substantial fraction (> 10%) of the observed variance in *F*_ST_ or branch length among the height GWA SNPs. This observation suggested that, at least in certain periods of human evolution, strong genetic covariance might have existed between height and fitness, which enabled rapid polygenic adaptation by allele frequency shifts at these height influencing variants so that a new phenotypic optimum could be reached to meet environmental challenge (Pritchard et al., 2010). Beyond this example, similar but less significant correlations between effect size and “strength of selection” were observed in GWA SNPs associated with serum urate level and diastolic blood pressure.

Polygenic adaptation is an inherently multivariate process and natural selection often acts upon sets of functionally related traits. Following the classical quantitative genetics mode, allele frequency changes in response to selection are determined by both selection gradients and genetic correlations between all the traits under selection (Blows, 2007, Lande, 1979, Lande and Arnold, 1983). Consequently, the observed strong correlation between the effect sizes of SNPs associated a phenotypic trait and the “strength of selection” strongly suggest the phenotypic trait (e.g. height) has been the primary target of selection and the allele frequency changes might have been driven by the selection pressure on the trait (“focal” trait). Whereas for those traits in which we did not observe any significant correlation between effect size and “strength of selection”, the selection might act through other traits with shared genetic components due to pleiotropy (Mackay et al., 2009).

Because the effect size on a “focal” trait (i.e. a trait with fitness effect) and the estimated “strength of selection” (i.e. *F*_ST_ or branch length) may not necessary follow a linear relationship, we used Pearson's correlation (for linear correlation) as well as Kendall's *tau* (for non-linear dependence) to access the statistical significance. Usually, the Kendall's *tau* reported more significant results (such as in SNPs associated with height and urate level), indicating the quantitative relationship between the effect size on a “focal” trait and the effect on fitness is complex and cannot be simply predicted by simple quantitative genetic models (Orr, 2009). We also observed examples in which the Pearson's correlations were more significant (such as in SNPs associated with DBP and platelet counts). However, it seems that the highly significant linear correlation was driven by a number of outliers (with both uncommonly large effect size and between population differentiation).

### 4.4 Signs of directional selection

We used three different methods to examine whether there were consistent signs of directional selection among the trait associated SNPs. The first two methods were based on the comparison of counts or allele frequency distributions between derived alleles with plus or minus effects. The third method took the advantages of the ancestral allele frequencies estimates from our ML branch length analysis and directly compared the allele frequency changes between current and ancestral populations. The validity of these methods depends on two assumptions: first, the mutant (derived) alleles have an equal chance to have either plus or minus effect on a complex trait and second, the detection rates of risk alleles with plus or minus effects were same by GWA studies. The first assumption was suggested by Fisher's infinitesimal model (Hill, 2010) and was supported by many studies (Gibson, 2011, Hill, 2010). The assumption of same detection rates of alleles with plus or minus effects is generally true in the association studies of quantitative traits with no sampling bias. But case/control studies of less common complex diseases might favor detection of low-frequent alleles that increase disease risk due to the enrichment of cases. Nevertheless, we did not observe significant evidence of directional selection among most of the traits examined in this study, except for adiponectin — most of the derived alleles (13 of14) of the associated SNPs reduce the adiponectin level (p-value = 0.0018).

Again, using height as an example, we did not observe systematic allele frequency changes that favor either increase or decrease of height in the three continental populations. These observations might suggest that, although the differentiation between populations was accelerated by selection forces, the trajectories or the directions of allele frequency changes were highly stochastic. This observation is in line with Amato et al. (2011), who suggested the behavior of frequency changes of alleles under (balancing) selection resembles a statistical mechanics phenomenon called Spontaneous Symmetry Breaking (SSB), in which case the allele frequencies of trait influencing alleles are randomly driven by selection in different populations and modified stochastically by the initial condition and demographic events. The overall outcome is an increase of diversity among populations (i.e. high *F*_ST_) but not predictable trajectories of allele frequency changes. However, the study by Turchin et al. (2012) demonstrated evidence of directional changes in allele frequencies of height SNPs between Northern Europeans and Southern Europeans, which might be governed by the deterministic force of directional selection (Fu and Akey, 2013).

Another possible explanation of this “drifting” (non-directional) pattern of allele frequency changes under selection could be the temporal or spatial fluctuation in fitness (Miura et al., 2013, Orr, 2009). For example, a genotype might enjoy high fitness and the allele frequency increases over a short time period or in a refined geographical region; but in another time period or region the same genotype might have low fitness and the frequency decreases. Over a longer time-scale or broader regions, the genotype might seem to be selectively “neutral” on average but the rate of frequency “drift” (changes without predictable directions) could be speeded-up by natural selection. This explanation might also answer why Turchin et al. (2012) detected directional changes in allele frequencies of height SNPs but we did not — because the European populations they studied represent a short time-period in human evolution and locate in refined geographical regions, in which the selection force might be more homogenous. Besides this possibility, stabilizing selection and assortative mating might also increase differentiation among populations (i.e. high *F*_ST_) but not necessarily lead to directional changes in allele frequencies.

Despite the fact that the power of our tests to detect directional selection was limited by the number of available GWA SNPs, we believe the negative results obtained from the analysis of directional changes might reflect a general fact that, allele frequency changes in response to natural selection of complex traits might be highly stochastic. That is, although collectively significant signatures of natural selection on a complex trait might be evident, the long-term response to selection might be unpredictable because a myriad of different selection mechanisms and various environmental challenges and demographic factors fueled the process. This conclusion might have an implication in complex disease genetics study: association mapping in different populations is likely to reveal different sets of trait associated SNPs with prominent evidence of population differentiation; however, the allele frequency differences in these trait associated SNPs may not follow a simple evolution model and cannot be mechanistically interpreted as the outcome of local adaption in different populations (i.e. disease prevalence). This conjecture was also supported by Southam et al. (2009), who did not find consistent patterns of selection among the T2D and obesity susceptibility variants to support the thrifty genotype hypothesis.

### 4.5 Individual SNPs under selection

Among the 1296 distinct SNPs associated with the 38 complex traits, we identified 36 SNPs with signatures of divergent selection as reflected by significantly higher global *F*_ST_ or total branch length at p-value < 0.01, reflecting an excess of hits expected by chance alone (Table 4). Nearly half of the identified SNPs showed significant iHS scores (Voight et al., 2006) (|iHS| > 2.0), and most of them were negative (iHS < − 2) in European or Asian populations, indicating the derived alleles are under selection in these two populations. Some of the significant SNPs associated with different complex traits are clustered together. For example: rs9388489 (T1D), rs1361108 (menarche, age at onset) and rs1490384 (height) near the *CENPW* gene; rs386000 (HDL) and rs103294 (prostate cancer) near the *LILRA3* gene; and multiple trait associated SNPs at the *SH2B3*-*ATXN2* locus were respectively in near perfect LD. This clustering pattern may indicate that some loci under selection have pleiotropic effects on multiple apparently unrelated complex traits (Sivakumaran et al., 2011, Wagner and Zhang, 2011). Alternatively, the extended LD between multiple trait associated variants at a locus might be a result of hitchhiking effect of a major selective sweep (McVean, 2007).

Some of the identified SNPs were in genomic loci with well-established evidence of natural selection, such as the *SH2B3*-*ATXN2* locus (Sabeti et al., 2006, Zhernakova et al., 2010) and the *OCA2*-*HERC2* locus (Donnelly et al., 2012). The *SH2B3*-*ATXN2* locus contains GWA SNPs associated with many complex traits and disorders, including various auto-immune disorders (Barrett et al., 2009, Dubois et al., 2010, Jin et al., 2012, Stahl et al., 2010), blood cell traits (Gieger et al., 2011, Gudbjartsson et al., 2009, van der Harst et al., 2012), blood pressure (International Consortium for Blood Pressure Genome-Wide Association, S., et al., 2011, Levy et al., 2009), urate level (Kottgen et al., 2013), cholesterol (Teslovich et al., 2010)) and CHD (Schunkert et al., 2011), etc. The reported GWA SNPs within this locus (rs3184504, rs4766578, rs653178 and rs11065987) are in close LD and the derived alleles of these SNPs formed a haplotype associated with increased risk to auto-immune disorders and increased blood pressure, urate level but reduced cholesterol. This haplotype of derived alleles is common in the European population but rare in Africans and Asians. Evolutionary analysis has suggested a recent selective sweep in *SH2B3* in the European populations in response to possible bacterial infection (Zhernakova et al., 2010). It should be noted that the haplotype of the *SH2B3*-*ATXN2* locus extends to the nearby *ALDH2* gene, which has also been suggested to be under selection (Oota et al., 2004). Genetic variants of *OCA2*-*HERC2* locus are associated with eye and hair pigmentation (Eiberg et al., 2008, Kayser et al., 2008, Liu et al., 2010, Sulem et al., 2007). The variant (rs1129038) associated with vitiligo is in close LD with the variants that associated with eye or hair color (i.e. rs12913832, rs916977 and rs1667394). These variants are common in European population but rare in African and Asian. A third example was a SNP associated with total cholesterol (rs7570971) in *RAB3GAP1* gene. This gene locates near *LCT* gene (Bersaglieri et al., 2004) and the derived allele (C) of rs7570971 that reduces cholesterol exists almost exclusively on the haplotype that is associated with lactase persistence, which is common in Europeans but rare and absent in African and Asian populations, respectively.

In addition to these examples, five SNPs associated (rs12103, rs7517810 (tagged by rs9286879), rs11150589, rs2472649, rs670523) with IBD or its subforms (i.e. Crohn's disease, ulcerative colitis (UC)) showed significant evidence of divergent selection (Table 4). These SNPs were found in the top 8 significant SNPs under directional selection (with p-value < 0.01) identified by Jostins et al. (Supplementary Table 5 of reference (Jostins et al., 2012)) and the ranking of significance of these SNPs were almost same between the two studies. Two of the other three significant loci reported by Jostins et al. (rs7608910/rs13003464 and rs6088765/rs2425019) were also located or near SNPs under significant selection identified by our study, although not reported as IBD associated SNPs: rs13003464 was listed as a Celiac disease associated SNP and rs6088765 was near the *UQCC/GDF5/CEP250* height associated region indexed by rs143384. Our results were also consistent with Ding and Kullo (2011), who studied differentiation in allele frequencies of SNPs associated with cardiovascular diseases. Most of the significant SNPs reported in their study were replicated in our study either among our top significant list (Table 4) or with marginal significance (p-value < 0.05). The consistency of the identified selection signals between our analysis and previous studies provides support to our approach and the power of using 1000 Genome data in detecting selection signatures. The per-variant selection data used by Jostins et al. (2012) were generated by the TreeMix (Pickrell and Pritchard, 2012), which measures the extent of differentiation of allele frequencies among populations, is similar to our branch length analysis. The study by Ding and Kullo (2011) was mainly based on *F*_ST_ analysis. However, both of these two previous studies used the Human Genetic Diversity Panel data from more than 50 populations, which is more diverse than the 1000 Genome data.

### 4.6 Limitations

It should be noted that the source data and our analytical approaches are subject to some limitations. First, our current study relies on the records of SNP-trait associations extracted from the GWA catalog (Hindorff et al., 2009). Although it contains the most comprehensive list of SNP associations of complex traits, the number and quality of the reported associations in the GWA catalog vary among published GWA studies. To assure the quality of extracted records, we only included GWA SNPs with stringent p-value (< 5 × 10^− 8^) reported by large studies (sample size > 1000). Because of the large number of extracted records (nearly 1500), we could not check the original reports to verify the accuracy of the records. More importantly, the reported GWA SNPs were mostly detected using commercial genotyping chips in samples usually of European origin and in combination the identified SNPs only explain a very small fraction of the phenotypic variance. Thus, the GWA SNPs may not provide a representative picture of the entire underlying genetic architecture of complex traits. Consequently, the selection signatures of complex traits detected in our current study are incomplete and probably biased in certain ways. For example, a negative result based on the known SNPs of a complex trait does not necessarily mean there is no polygenic adaptation of the trait. With the accumulation of extended sets of GWA SNPs of complex traits by larger studies in diverse populations, we will obtain a more complete picture of polygenic adaption of complex traits.

Second, our approaches to detect natural selection were based on the comparison of allele frequency differences between populations and therefore cannot capture signals of selection before the human migration out of Africa (50,000 to 75,000 years ago) (Sabeti et al., 2006). Future investigation using other statistical methods (Biswas and Akey, 2006, Hohenlohe et al., 2010, Nielsen et al., 2007, Sabeti et al., 2006) might help identify selection signatures on different time scales or at different stages of a selective sweep. Single-locus estimates of population differentiation (either *F*_ST_ or branch length) are highly uncertain, which might lead to false discoveries of selection signature (Holsinger and Weir, 2009, Weir et al., 2005). Recent studies (Elhaik, 2012, Maruki et al., 2012) and our results (not shown) suggested the empirical distribution of population differentiation estimates are substantially influenced by allele frequency. Therefore, we selected “null” SNPs with matched ancestral allele frequency to the tested GWA SNPs on a one-by-one basis in obtaining the empirical p-values.

Third, we implicitly assumed the reported GWA SNPs were the real functional variants. It is always possible that a reported SNP is simply in close LD (high *r*^2^ and hence similar allele frequency) with the real functional variant in the discovery samples but not in other populations. Such different LD patterns between populations would likely lead to inaccurate estimates of population differentiation and hence the signature of natural selection. Allelic heterogeneity at the complex trait associated loci (Lango Allen et al., 2010, Zhang et al., 2012) may further complicate detection and interpretation of natural selection signals.

Lastly, we used a purely empirical method to access the statistical significance of identified signatures of natural selection. This approach does not require assumptions about population demography and simply considers “outliers” as likely targets of natural selection (Akey et al., 2002). Simulation study suggested that such methods have a high false-negative rate and tends to provide a biased view of selection, depending on the mode of selection and demographic history (Nielsen et al., 2007, Teshima et al., 2006). In addition, we did not rigidly account for multiple testing in our study. Therefore, the “significant” results from our study should be interpreted as exploratory rather than statistically confirmatory evidence.

## 5 Conclusions

We present here a comprehensive empirical study of natural selection on standing variants associated with complex human traits. Although the known GWA SNPs represent incomplete genetic architecture of the complex traits and our methods might suffer from some technical limitations, our results revealed some general features of natural selection on complex traits associated variants. First, natural selection on standing variants associated with complex traits is a common phenomenon, although the strength of selection is usually weak and hard to be robustly detected for individual variants. Second, the signatures of selection of a particular trait usually enriched in specific human lineages indicating recent adaption of different complex traits might be associated with temporarily and geographically specific environmental challenges. Third, as indicated by the strong correlation between the effect sizes and the estimated strength of selection, some traits (e.g. height or urate level) might have strong fitness effect and might be the “focal” polygenic adaptive traits, through which selection drives the changes in allele frequencies. But for those traits without clear correlation between the effect sizes and the strength of selection, the selection on the trait associated variants might act through other correlated traits. Fourth, the genetic response (i.e. changes in allele frequencies) to natural selection of complex traits could be highly stochastic. That is, polygenic adaptation can accelerate differentiation between populations but generally does not produce predictable directional changes in allele frequencies. Fifth, the evolutionary scenario of a single complex trait could be very heterogeneous and multiple mechanisms (i.e. pleiotropy, hitchhiking, epistasis, etc) may act together on different variants.

Taken together, natural selection on standing variants of complex traits is a common and complex phenomenon governed by a wide range of evolutionary factors, but none with clear dominant and deterministic effect. To pinpoint the genetic changes in responding a particular environmental challenge and the identification of underlie adaptive traits will be a challenging task even with large genomic data sets.

## Figures and Tables

**Table 1:** Different measures of differentiation in allele frequencies among populations.

Measure	Description
*F_ST_*	
Global	Global *F*_ST_ calculated from 9 populations
AFR_ASN	Average *F*_ST_ between major continental populations
AFR_EUR	
ASN_EUR	
AFR	Average *F*_ST_ within continental populations
ASN	
EUR	
Branch length	
Total	Sum of all branch lengths
AFR	Major split to African populations
EUA	Major split to Eurasia
ASN	Asia split from Eurasia
EUR	European split from Eurasia
AFRs	Average branch length of Afrian populations
ASNs	Average branch length of Asian populations
EURs	Average branch length of European populations

**Table 2:** The 38 complex traits/diseases.

Trait	Total^a^			Selected^b^		Effect^c^	
	records	snps	pubs	records	snps	snps	PMID
*Physical traits*							
Height	425	385	19	265	178	167	20881960
Body mass index	112	102	16	64	35	30	20935630
Systolic blood pressure	30	29	3	25	23	17	21909115
Diastolic blood pressure	32	30	3	28	22	16	21909115

*Lipids*							
Cholesterol, total	67	65	2	61	50	44	20686565
LDL cholesterol	104	76	13	85	36	29	20686565
HDL cholesterol	119	96	12	104	47	44	20686565
Triglycerides	98	78	11	83	33	30	20686565

*Other metabolites and physiological traits*							
Urate levels	70	63	8	32	29	29	23263486
C-reactive protein	54	46	8	33	18	17	21300955
Adiponectin levels	48	44	9	28	16	14	22479202
Liver enzyme levels (gamma-glutamyl transferase)	26	26	1	25	25	25	22001757
Platelet counts	74	70	3	55	53	53	22139419
Ventricular conduction	25	25	1	23	23	23	21076409
QT interval	74	68	7	34	18	13	19305408
Corneal structure	32	31	3	27	27	25	23291589

*Inflammatory and autoimmune disorders*							
Inflammatory bowel disease	124	118	4	116	108	107	23128233
Crohn's disease	212	172	14	168	95	65	21102463
Ulcerative colitis	129	101	8	92	57	41	21297633
Celiac disease	50	43	3	32	27	16	20190752
Systemic lupus erythematosus	111	90	12	52	29	16	19838193
Rheumatoid arthritis	96	78	14	42	28	15	20453842
Psoriasis	44	36	7	22	16	7	20953190
Multiple sclerosis	166	146	15	60	48	45	21833088
Type 1 diabetes	97	78	10	60	39	8	17554260
Primary biliary cirrhosis	41	32	4	21	17	14	21399635
Vitiligo	37	37	6	27	25	12	22561518

*Common diseases*							
Type 2 diabetes	212	140	34	86	49	21	20581827
Coronary heart disease	150	127	20	55	35	24	21378990
Asthma	64	53	23	30	20	10	21804548
Parkinson's disease	84	67	15	32	16	9	21292315

*Mental disorders*							
Schizophrenia	129	111	28	67	49	9	21926974
Bipolar disorder	128	115	19	56	42	15	21926972

*Cancers*							
Breast cancer	76	61	24	33	19	8	20453838
Prostate cancer	116	82	21	70	34	9	18264097
Colorectal cancer	66	47	18	27	21	5	20972440

*Menstruation related*							
Menarche (age at onset)	47	44	5	37	33	33	21102462
Menopause (age at onset)	40	34	3	23	18	17	22267201

a The number of association records (records), SNPs (snps) and publications (pubs) in GWA catalog.

b The number of records and SNPs that passed our selection criteria.

c The number of SNPs with effect size information (estimation and direction) and the PMID of the source article.

**Table 3:** Empirical p-values of the mean *F*_ST_ and branch length measures estimated from the GWA SNPs.

Trait	Number of SNPs							Empirical p-values of *F*ST measures			Empirical p-values of branch length measures							
			Global	Average	AFR_ASN AFR_EUR ASN_EUR			AFRs	ASNs	EURs	Total	AFR	EUA	ASN	EUR	AFRs	ASNs	EURs
*Physical traits*																		
Height	178	167	**0.0088**	**0.004**	0.0336	**0.0004**	0.283	0.799	0.111	0.713	**0.0092**	**0.0004**	0.0428	0.657	0.0808	0.664	0.1	0.713
Body mass index	35	30	0.326	0.618	0.645	0.288	0.301	0.676	0.912	0.823	0.588	0.328	0.762	0.276	0.527	0.823	0.882	0.803
Systolic blood pressure	23	17	0.341	0.608	0.77	0.446	0.141	0.771	0.877	**0.0022**	0.27	0.686	0.715	0.315	0.05	0.646	0.787	**0.0036**
Diastolic blood pressure	22	16	0.554	0.798	0.944	0.611	0.2	0.876	0.632	**0.0054**	0.581	0.919	0.861	0.384	0.103	0.794	0.656	**0.0082**

*Lipids*																		
Cholesterol, total	50	44	0.219	0.731	0.241	0.317	0.459	0.296	0.164	**0.0032**	0.111	0.0964	0.425	0.435	0.599	0.419	0.163	**0.002**
LDL cholesterol	36	29	0.679	0.807	0.541	0.773	0.471	0.128	0.089	0.953	0.678	0.439	0.738	0.552	0.596	0.2	0.148	0.986
HDL cholesterol	47	44	0.233	0.256	0.389	0.668	0.0732	0.531	0.094	0.158	0.28	0.302	0.881	0.136	0.331	0.473	0.117	0.22
Triglycerides	33	30	**0.0094**	0.0146	**0.0042**	0.168	0.179	0.0382	**0.0032**	0.409	**0.0016**	**0.0072**	0.0304	0.0374	0.866	0.0386	0.0126	0.342

*Metabolites and physiological traits*																		
Urate levels	29	29	**0.0082**	0.0226	0.117	0.073	0.0226	0.953	0.643	0.0348	0.0204	0.266	0.357	0.0192	0.042	0.925	0.658	0.032
C-reactive protein	18	17	0.366	0.168	0.335	0.409	0.285	0.0294	0.325	0.0586	0.349	0.487	0.549	0.282	0.583	0.0684	0.522	0.081
Adiponectin levels	16	14	0.887	0.971	0.629	0.992	0.679	0.496	0.88	0.26	0.929	0.9	0.877	0.667	0.761	0.446	0.692	0.177
Ggamma-glutamyl transferase	25	25	0.294	0.411	0.136	0.626	0.301	0.0402	0.434	0.184	0.259	0.298	0.501	0.149	0.788	0.0714	0.377	0.218
Platelet counts	53	53	0.618	0.859	0.434	0.332	0.884	0.438	0.0784	0.277	0.348	0.0576	0.595	0.748	0.845	0.458	0.0224	0.307
Ventricular conduction	23	23	0.772	0.827	0.904	0.717	0.653	0.149	0.239	0.109	0.787	0.841	0.813	0.768	0.532	0.137	0.165	0.113
QT interval	18	13	0.294	0.23	0.904	0.0896	0.243	0.598	0.136	0.939	0.553	0.483	0.668	0.542	0.214	0.539	0.179	0.952
Corneal structure	27	25	0.37	0.32	0.36	0.324	0.45	0.516	0.777	**0.0024**	0.336	0.517	0.268	0.439	0.659	0.286	0.808	**0.0024**

*Inflammatory and autoimmune disorders*																		
Inflammatory bowel disease	108	107	0.122	0.122	0.232	0.127	0.33	0.237	0.15	0.164	0.089	0.0764	0.384	0.201	0.451	0.311	0.196	0.298
Crohn's disease	95	65	0.0898	0.178	0.556	0.299	0.0188	0.197	0.113	0.032	0.131	0.643	0.935	0.0154	0.0654	0.176	0.0824	0.18
Ulcerative colitis	57	41	0.478	0.457	0.268	0.91	0.125	0.584	0.0608	0.58	0.437	0.641	0.84	0.0674	0.506	0.618	0.0608	0.68
Celiac disease	27	16	0.141	0.186	0.55	0.0352	0.177	0.708	0.658	0.805	0.125	0.0622	0.566	0.123	0.215	0.649	0.723	0.825
Systemic lupus erythematosus	29	16	**0.008**	0.0554	0.0328	**0.0028**	0.396	0.367	0.292	0.27	**0.0072**	0.0268	**0.001**	0.322	0.559	0.322	0.199	0.217
Rheumatoid arthritis	28	15	0.399	0.415	0.264	0.719	0.299	0.375	0.0542	0.336	0.42	0.388	0.717	0.422	0.419	0.324	0.115	0.297
Psoriasis	16	7	0.0952	0.167	0.087	0.167	0.462	0.218	0.0842	0.581	0.215	0.099	0.347	0.413	0.707	0.159	0.147	0.586
Multiple sclerosis	48	45	0.707	0.472	0.341	0.887	0.422	0.626	0.0212	0.258	0.585	0.857	0.505	0.465	0.462	0.681	0.038	0.292
Type 1 diabetes	39	8	0.0458	0.0994	0.528	0.0956	0.013	0.18	0.107	0.167	0.0334	0.423	0.408	0.0918	0.0248	0.104	0.0318	0.174
Primary biliary cirrhosis	17	14	0.18	0.199	0.156	0.495	0.306	0.823	0.272	0.0652	0.143	0.0694	0.777	0.08	0.742	0.78	0.18	0.0206
Vitiligo	25	12	0.0262	0.642	0.812	0.0234	0.0394	0.955	0.197	**0.0036**	**0.0086**	0.269	0.344	0.0808	**0.0046**	0.839	0.0734	**0.0028**

*Common diseases*																		
Type 2 diabetes	49	21	0.0354	0.151	0.221	0.121	0.0894	0.508	0.32	0.332	0.0264	0.404	0.0386	0.103	0.0672	0.354	0.395	0.279
Coronary heart disease	35	24	0.133	0.129	0.468	0.0338	0.385	0.601	0.321	0.103	0.0712	0.0416	0.233	0.554	0.266	0.775	0.099	0.0996
Asthma	20	10	0.225	0.194	0.36	0.499	0.148	0.951	0.733	0.147	0.269	0.243	0.729	0.155	0.235	0.929	0.738	0.132
Parkinson's disease	16	9	0.804	0.899	0.918	0.571	0.556	0.763	0.288	0.0482	0.637	0.719	0.793	0.621	0.459	0.695	0.0644	0.043

*Mental disorders*																		
Schizophrenia	49	9	0.0164	0.0114	**0.0048**	**0.0026**	0.556	0.31	0.297	0.381	**0.0068**	**0.0004**	0.0258	0.557	0.606	0.289	0.293	0.333
Bipolar disorder	42	15	0.63	0.595	0.758	0.707	0.448	0.348	0.144	0.0112	0.582	0.803	0.822	0.689	0.269	0.224	0.044	0.0264

*Cancers*																		
Breast cancer	19	8	0.298	0.233	0.253	0.607	0.18	0.708	0.434	0.949	0.364	0.645	0.506	0.0756	0.299	0.731	0.454	0.919
Prostate cancer	34	9	0.0174	0.0296	0.045	**0.0036**	0.449	0.777	0.042	0.405	0.01	**0.0002**	0.119	0.38	0.682	0.717	0.0176	0.24
Colorectal cancer	21	5	0.234	0.165	0.259	0.257	0.385	0.0662	**0.0006**	0.096	0.0648	0.171	0.225	0.261	0.429	0.109	0.0104	0.143

Menstruation related																		
Menarche (age at onset)	33	33	0.828	0.801	0.715	0.924	0.461	0.905	0.615	0.605	0.799	0.851	0.697	0.567	0.231	0.954	0.659	0.696
Menopause (age at onset)	18	17	0.324	0.218	0.451	0.24	0.256	0.837	0.848	0.236	0.402	0.52	0.271	0.378	0.358	0.829	0.634	0.352

* Significant p-values (< 0.01) are highlighted in bold.

**Table 4:**
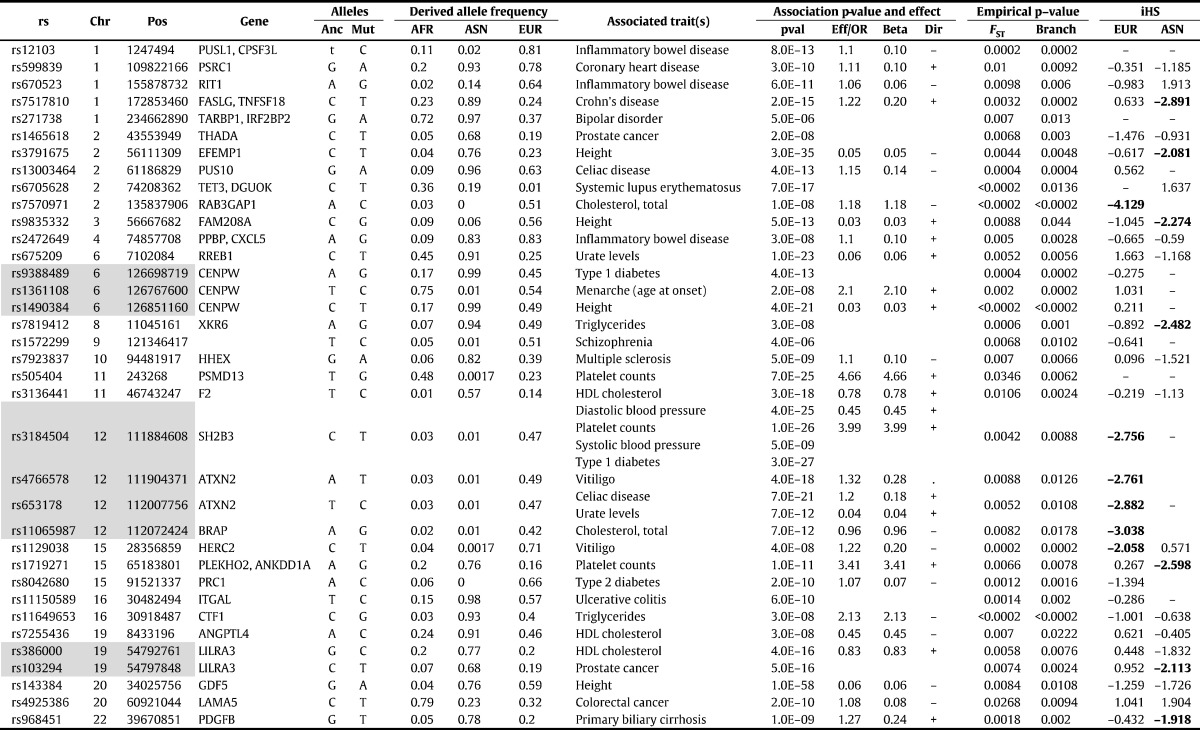
List of GWA SNPs with top significant signals of accelerated differentiation.

Note: SNPs in close LD are highlighted in gray blocks. Significant iHS scores (|iHS| > 2.0) are printed in bold.
